# Unavoidable Risks: Local Perspectives on Water Contact Behavior and Implications for Schistosomiasis Control in an Agricultural Region of Northern Senegal

**DOI:** 10.4269/ajtmh.19-0099

**Published:** 2019-08-26

**Authors:** Andrea J. Lund, Mouhamadou Moustapha Sam, Alioune Badara Sy, Omar W. Sow, Sofia Ali, Susanne H. Sokolow, Sylvia Bereknyei Merrell, Janine Bruce, Nicolas Jouanard, Simon Senghor, Gilles Riveau, David Lopez-Carr, Giulio A. De Leo

**Affiliations:** 1 Emmett Interdisciplinary Program in Environment and Resources, Stanford University, Stanford, California;; 2 Centre de Recherche Biomédicale – Espoir Pour la Santé, Saint Louis, Sénégal;; 3 Stanford University, Stanford, California;; 4 Hopkins Marine Station, Stanford University, Pacific Grove, California;; 5 Department of Surgery, Stanford Surgery Policy Improvement Research & Education Center (S-SPIRE), School of Medicine, Stanford University, Stanford, California;; 6 Pediatric Advocacy Program, Department of Pediatrics, School of Medicine, Stanford University, Stanford, California;; 7 Station d’Innovation Aquacole, Saint Louis, Senegal;; 8 Department of Geography, University of California, Santa Barbara, Santa Barbara, California

## Abstract

Human schistosomiasis is a snail-borne parasitic disease affecting more than 200 million people worldwide. Direct contact with snail-infested freshwater is the primary route of exposure. Water management infrastructure, including dams and irrigation schemes, expands snail habitat, increasing the risk across the landscape. The Diama Dam, built on the lower basin of the Senegal River to prevent saltwater intrusion and promote year-round agriculture in the drought-prone Sahel, is a paradigmatic case. Since dam completion in 1986, the rural population—whose livelihoods rely mostly on agriculture—has suffered high rates of schistosome infection. The region remains one of the most hyperendemic regions in the world. Because of the convergence between livelihoods and environmental conditions favorable to transmission, schistosomiasis is considered an illustrative case of a disease-driven poverty trap (DDPT). The literature to date on the topic, however, remains largely theoretical. With qualitative data generated from 12 focus groups in four villages, we conducted team-based theme analysis to investigate how perception of schistosomiasis risk and reported preventive behaviors may suggest the presence of a DDPT. Our analysis reveals three key findings: 1) rural villagers understand schistosomiasis risk (i.e., where and when infections occur), 2) accordingly, they adopt some preventive behaviors, but ultimately, 3) exposure persists, because of circumstances characteristic of rural livelihoods. These findings highlight the capacity of local populations to participate actively in schistosomiasis control programs and the limitations of widespread drug treatment campaigns. Interventions that target the environmental reservoir of disease may provide opportunities to reduce exposure while maintaining resource-dependent livelihoods.

## INTRODUCTION

The links between poverty and infectious disease are well known,^1^ particularly for the diverse group of neglected tropical diseases (NTDs). Neglected tropical diseases afflict more than one billion people worldwide, most of whom live in poverty.^2^ Among the NTDs is schistosomiasis, a disease caused by a parasitic worm of the genus *Schistosoma* and transmitted to humans by freshwater snails that serve as the parasite’s intermediate host. Schistosomiasis is widespread across tropical latitudes and affects an estimated 200 million people worldwide, with the vast majority of those cases occurring in sub-Saharan Africa.^3^ Transmission occurs when intermediate host snails release infective larvae into freshwater. People become infected by simply being in contact with that water, where free-swimming parasite larvae burrow directly into their skin. Such water contact is highly socially patterned and often dictated by economic needs.^4,5^

Clinically, schistosomiasis morbidity is characterized by chronic inflammation, often with nonspecific symptoms, including abdominal pain, diarrhea, or, in the case of urogenital schistosomiasis, hematuria.^6^ In situations of both acute and chronic infections, parasite eggs cause granulomatous lesions to develop in tissue. Years of chronic infection can lead to severe disease, including anemia, permanent organ damage, portal hypertension, liver fibrosis, and among those with urogenital schistosomiasis, bladder cancer and infertility in women.^6–8^ Morbidity due to chronic infection is often described as “subtle,”^9^ but has more recently been recognized as a sign of systemic disability with long-term consequences.^10,11^ Several observational studies suggest that chronic schistosomiasis can impact important elements of human capital—such as cognitive development and labor productivity—affecting economic development.^12–15^

Since becoming widely available in the 1980s, the antiparasitic drug praziquantel has been the centerpiece of schistosomiasis control, largely displacing the chemical snail control campaigns of the preceding decades.^16,17^ Mass drug administration (MDA) campaigns relieve people of existing infections, but do not address the environmental source of infection, nor the socioeconomic conditions that can perpetuate exposure. Reinfection can occur rapidly after treatment and operational research on program implementation has shown limited impact of MDA in some settings, where prevalence and intensity of infection remain high despite multiple rounds of treatment.^18,19^ These locations—known as persistent hot spots—likely experience high rates of reinfection following treatment because of a high force of transmission in the environment, arising from ecological characteristics of the snail population as well as human water contact behaviors.^19,20^

The ecological characteristics that lead to persistent schistosomiasis transmission may be related to the active management of water resources for agricultural and other economic needs. Infection rates are often elevated in close proximity to dams and irrigation schemes.^21,22^ Such landscape transformation increases the suitable habitat available to the parasite’s intermediate host snails.^23,24^ This occurred on the Senegal River, which forms the border between Senegal and Mauritania. A saltwater barrier at the mouth of the river, the Diama Dam, was designed to promote year-round agriculture in a region prone to drought. Before dam construction in 1986, limited, seasonal transmission of urogenital schistosomiasis occurred.^22^ A dramatic increase in prevalence and intensity of infection for both urogenital and intestinal schistosomiasis followed dam construction.^25,26^ Infection rates remain high today, making the region one of the most hyperendemic regions in the world, despite yearly school-based MDA since 1999 through the National Schistosomiasis Control Program.^27^ These anthropogenic environmental changes have created a “pathogenic landscape”^28^ for the rapid spread of schistosomiasis that has so far resisted MDA-based control efforts.

Although MDA remains the primary schistosomiasis control strategy across sub-Saharan Africa, the existence of persistent hot spots across the continent makes clear the need for interventions that disrupt schistosome transmission in the environment.^18,19^ Historically, snail control has played a role in many successful schistosomiasis elimination campaigns.^17,29^ Since 2012, the World Health Assembly has promoted the integration of MDA campaigns with strategies focused on improving the environment and preventing new infections (ref. 30, p. 21). Such strategies include chemical and biological snail control^29,31–33^ as well as water, sanitation, and hygiene (WASH) interventions to reduce exposure to and contamination of the environment.^34–36^

The purpose of this study was to understand the experience of living in a hyperendemic landscape, seeking rural villagers’ perceptions of the freshwater environment and schistosomiasis risk in villages in northern Senegal, where agricultural livelihoods are common and the burden of schistosomiasis is high. We use qualitative data derived from focus group discussions with men, women, and youth living in the lower basin of the Senegal River to determine how local perceptions of the disease, its environmental source, and reported preventive behaviors are influenced by socioeconomic and environmental conditions. We consider how local efforts to reduce exposure and prevent infection are hampered by reinforcing feedbacks between poverty and disease. Finally, we discuss the use of local knowledge for designing sustainable interventions in schistosomiasis control programs and policies.

## METHODS

**Figure 1.**
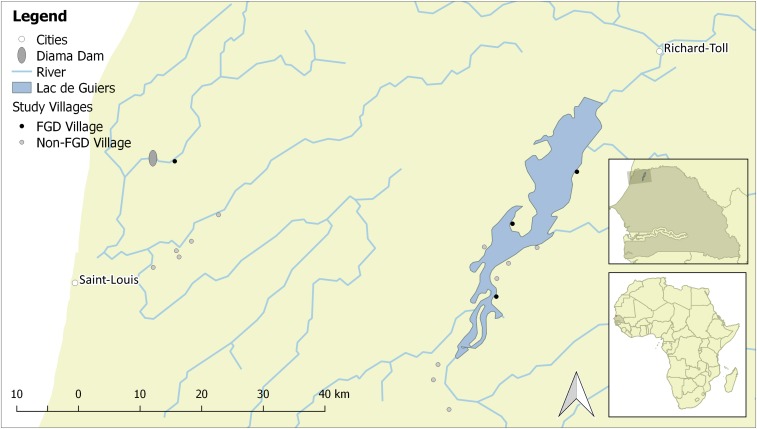
The lower basin of the Senegal River, including the Diama Dam and four study sites. This figure appears in color at www.ajtmh.org.

This qualitative study was a part of a larger ongoing study of socioecological determinants of schistosomiasis in the region.^37–40^ For this larger study, 16 villages were chosen to represent the rural, high-transmission sites common in the region (Figure 1). The villages in the sample frame meet the following criteria: 1) close proximity to the Senegal River, its tributaries, or the Lac de Guiers, 2) at least one but no more than four regularly used freshwater access sites, 3) not in the top or bottom 10th percentiles of human population density, 5) had a school with at least 30 students in grades 1–3, 5) a nonzero prevalence of hematuria, an indicator of urogenital schistosome infection and 6) were accessible by truck in the rainy season.

Of these 16 villages in the larger study, four were chosen for further qualitative data collection through focus group discussions. This subset of villages was selected based on environmental and geographic characteristics to understand both the nuance and complexity of water contact behavior in the region. Villages were selected to represent the dynamics on both the Senegal River and its tributaries as well as the Lac de Guiers and were approximately proportional to the geographical distribution of these villages in the larger study: one on the river and three on the lake (Figure 1). Beyond this stratification, the complexity of water contact behavior was captured by selecting villages that had multiple access points. Maka Diama (hereafter, River Village R) was located along the Senegal River, where irrigation infrastructure supporting the cultivation of rice was constructed in conjunction with the Diama Dam. The three remaining villages—Diokhor (Lake Village W), Mbane (Lake Village E1) and Syer (Lake Village E2)—were located on the shores of Lac de Guiers, where irrigated agriculture is undertaken on a smaller scale and irrigation infrastructure comprised hand-dug, earthen canals (Figure 1).

The schistosomiasis burden among school-aged children in this setting is high. Prevalence of urogenital schistosomiasis exceeds 75% across all 16 villages and exceeds 85% in all four focus group villages. The prevalence of intestinal schistosomiasis is lower but still considerable: 32.3% across all 16 villages and ranging from 13% to 32% in focus group villages (Table 2). All four focus group villages are rural, located at least at a 30-minute drive from the nearest market town (Figure 1). Reported occupations among adults are primarily agricultural (Table 2). Access to piped water in the focus group villages varies substantially from 17% in Lake Village W to 100% in Lake Village E1, but to varying degrees, households (HHs) in all four villages rely on surface water for domestic chores such as laundry (Table 2).

**Table 1:** Focus group characteristics by village and demographic group

Village	Men		Women		Youth		Total	
	*N*	Ages (years)	*N*	Ages (years)	*N*	Ages (years)	*N*	Ages (years)
River	6	20–53	8	23–60	6	12–15	20	12–60
Lake W	4	47–70	6	32–68	7	10–15	17	10–70
Lake E1	6	34–49	6	27–41	5	10–14	17	10–49
Lake E2	9	27–68	6	18–32	5	10–15	20	10–68
Total	25	20–70	26	18–68	23	10–15	74	10–70

A field team of one U.S.-based research (A. J. L., female) and two Senegalese-based research assistants (M. M. S. and A. B. S., both male) designed and conducted focus group discussions in the phenomenological qualitative inquiry to better understand how people perceive and interact with their environment.^41^ Focus group discussions within the four villages were performed concurrently during the baseline data collection of HH surveys for the larger study. Data collection for both studies occurred in August 2016. Three group discussions in each village represent the distinct perspective of three demographic groups: 1) adult men, 2) adult women, and 3) mixed gender youth, aged 10–18 years. The focus groups were conducted separately to facilitate open conversation among peer groups as well as to maximize detail and nuance expressed when describing perspectives on water contact behavior, which are known to vary by age and gender.^4^

On arrival, in each village, the research team spoke with a key informant, such as the village chief or a community health worker, to explain the objectives of the study. The key informant was asked to recruit five to six individual participants who had diverse perspectives and interactions with the environment for each of the three groups. The key informant then gathered available villagers, organizing them according to the age and gender criteria outlined by the research team. Relationships with key informants in each village are maintained by personnel at a biomedical research center, *Centre de Recherche Biomédicale-Espoir Pour La Santé* (CRB-EPLS), located in Saint-Louis (Figure 1). Because of existing relationships between key informants and CRB-EPLS personnel, the research team was known to be visiting the village to gather information about schistosomiasis.

Focus groups lasted, on average, 1 hour and 20 minutes. Discussions were conducted in semi-private locations, where participants could speak freely with minimal distraction and ambient noise. Given the collective nature of Senegalese culture, however, it was often impractical to ensure total privacy. Discussions were held within the home of a community leader, often the chief or other key informant for a given village, to maximize privacy. The research team was trained in focus group moderation. Each discussion was moderated according to a structured guide, including the following key prompts: 1) identify and describe the different places where people come into contact with water, 2) how people interact with those places, and 3) how people perceive schistosomiasis risk at each of those places (Supplemental Appendix 2). Group discussions were led by a moderator and audio-recorded in the local language Wolof. A bilingual Wolof- and French-speaking notetaker recorded notes in French. These notes were complemented by debriefing notes following each discussion. Audio recordings were transcribed in French by the moderator and notetaker. For terms that were difficult to directly translate from Wolof, transcriptionists kept the Wolof term in the transcript with a footnote explaining its meaning in French.

Transcripts were subsequently iteratively coded in French by two coders (A. J. L. and O. W. S.) using Dedoose (Sociocultural Research Consultants, LLC, Los Angeles, CA), a web-based application for qualitative and mixed methods data analysis. Group characteristics, such as age-group (adult or youth) and gender (male, female, or mixed) were used to conduct subsequent analyses. The preliminary codebook was developed inductively by the lead author: rather than relying on a priori assumptions about the text, coding categories were generated based on reading two of the 12 transcripts that covered gender and geographic diversity in the sample.^42^

The second coder (O. W. S.), a Senegalese citizen and native French speaker, was trained by the lead author using the preliminary codebook. This pair co-coded six of the 12 transcripts, meeting to adjudicate discrepancies in each one. Through these adjudication discussions, coding guidelines were iteratively adjusted after reaching 100% agreement, and revisions were made to the codebook and code definitions as the team detected additional nuance in the data.^42^ The native French speaker solo-coded the remaining six transcripts with the second coder spot-checking coded excerpts for consistency in code application and interpretation.

Once coding was complete, theme analysis—or recognition of patterns in the data—was conducted on the coded transcripts by the lead author with input from the second coder.^43,44^ The theme analysis process included examining excerpts by code, noting repetitions, meaningful relationships and conceptual links across different coding categories, and demographic and geographic perspectives. Based on codes that frequently co-occurred, the links between codes were mapped visually by the two coders and codes commonly linked in this map formed preliminary themes. This organizational process facilitated the construction of quote tables by preliminary theme across demographic diversity in the sample.

The extent to which each emergent theme was represented across the gender, age, and geographic diversity of the sample was determined by a simple tally system (Supplemental Tables 1–3). Any theme that occurred in focus groups with men, women, and youth as well as in three or four of the sampled villages was considered sufficiently common that it was appropriate to talk about these findings generally across the sample. Preliminary findings were presented to a subset of participants in each group during a subsequent field trip in July 2018 to validate findings.^41^ Findings were reinforced and modified accordingly.

The study received approval from the National Committee of Ethics for Health Research from the Republic of Senegal (Protocol #002659/OHRP) as well as the Institutional Review Boards of the University of California, Santa Barbara (Protocol #19-17-0676), and Stanford University (Protocol #43130). All focus group participants provided verbal informed consent.

## RESULTS

This study collected perspectives from a total of 74 individual participants (25 men, 26 women, and 23 youth) in 12 focus group discussions in the four villages in our sample frame (Table 1). Focus groups ranged in size from four to nine participants each. All four focus group villages—which range in size, proportion of people engaged in agriculture, and access to piped water—experience intense transmission of schistosomiasis that is tied to the environmental change in the region, resulting from dam construction (Table 2).

**Table 2:** Sociodemographic characteristics of all villages in larger study (*n* = 16 villages) as well as the four villages where focus group discussions were conducted, derived from HH survey data collected in August 2016

		Village			
	Overall	River R	Lake W	Lake E1	Lake E2
Total people	10,970	642	1,067	1,368	578
% Wolof ethnicity	72	65	89	90	88
Total adults	5,824	338	456	618	231
% Cultivators	27.4	23.1	47.6	50.8	53.2
% Homemakers	27.1	29.6	32.0	31.9	32.0
% Traders	10.2	11.2	11.2	9.1	12.1
% Fishers	4.6	3.6	3.7	1.5	12.6
% Day laborers	13.9	6.2	2.9	0.8	0.4
% Artisans	0.8	1.2	0.2	0.0	1.3
Total SAC	1,480	96	122	103	125
Urogenital prevalence in SAC (%)*	77.2	88.5	90.2	85.4	96.8
Intestinal prevalence in SAC (%)*	32.2	32.3	13.9	17.5	22.4
Total HH	663	43	47	57	36
Cultivated hectares	1,252	156	148	141	94.6
% HH with piped water access	77.4	97.7	17.0	100.0	11.1
% HH using surface water for drinking	11.4	2.3	83.0	0.0	83.3
% HH using surface water for laundry	24.6	58.1	97.9	14.0	72.2

HH = households; SAC = school-aged children.

* Prevalence data were collected as part of the larger 16-village study in February–April 2016.

Focus group discussions yielded three key findings. First, many residents, including those in positions of authority, have become knowledgeable about the environmental nature of schistosomiasis infection risk. Second, with this knowledge, residents of these villages have developed strategies to minimize their exposure to the parasite, at both the individual and community levels. And third, despite this knowledge and the willingness to act, several circumstances of daily life in this rural, resource-poor context limit the capacity of local people to reduce their exposure to the parasites. In the following paragraphs, we discuss the findings in depth.

### Finding 1: high local knowledge of schistosomiasis risk.

Focus group participants across the sampled age, gender, and geographic diversity expressed a basic understanding of schistosomiasis risk, commonly articulating comprehension that it is spread through water. One man from the River Village asserted that:

It is there [at the river] that bilharzia [schistosomiasis] enters your body. If you are told something else, know that you are cheated. It is there that you feel bilharzia enter squarely into your body. (53-year-old male gardener, River Village)

Participants noted that even children maintained an awareness of the environmental risk of schistosomiasis, associating risk of infection with specific water contact activities (Supplemental Table 1). They described the disease as ubiquitous in the environment and signaled recognition not only that the parasites are present in the environment but also that infection results from common water contact activities. Many residents of these villages clearly understood the mode of parasite transmission and noted that direct contact with surface water through a multitude of common water contact activities is how people become infected with schistosomiasis.

Although no participant in any of the focus group discussions referred to specific biological details of the schistosomiasis transmission cycle, such as the role of freshwater snails as the parasite’s obligate intermediate host, many did articulate an awareness of the presence of increased risk at midday. Across multiple villages and demographic groups, participants conveyed their perception that risk of infection increased when water contact occurred during midday, attributing the perceived increase in risk to high temperatures. A young man from Lake Village E2 said the following:

During the heat we can become infected with bilharzia [schistosomiasis] if we go to these three water points around noon and 1pm… During the heat… when the sun is at its zenith. (15-year-old male gardener, Lake Village E2)

This young man associated increased risk of schistosomiasis infection with the heat of midday. Although some participants articulated perceptions of increased risk at other times of day, either early in the morning or late in the afternoon, it was more common to associate increased risk with midday heat. Similar to the general awareness of the presence of schistosomiasis in the environment and the recognition of water contact as the mode of transmission, awareness of the daily cycles in schistosomiasis risk was expressed across gender, age, and geographic groups (Supplemental Table 1). The occurrence of this perception across most of the focus group discussions suggests that this scientifically recognized temporal dynamic of schistosomiasis risk may be common knowledge among residents of endemic villages in this region.

During discussions, focus group participants were prompted to talk about water contact sites in terms of whether it was acceptable to go to the bathroom near them. In their responses, participants frequently talked about how urination and defecation near the river or lake were forbidden, but they often qualified those statements, saying that it was still a relatively common practice. A 68-year-old man from Lake Village E2 spoke of it in terms of social accountability,

“If you want to go to the bathroom, we take the water from the canal and we hide… to do what we need… If someone sees you doing your thing in the lake, he tells you not to do it because you drink the water” (68-year-old male gardener, Lake Village E2).

It is clear that there are social rules and expectations around open urination and defecation. Hiding was commonly mentioned as a way to avoid being reprimanded by another villager. This sense also extended to the children, as stated by a woman in Lake Village E1,

“At all the [water access sites], you know, sometimes the children, they go there [to go to the bathroom] … when they are alone, they do what they want… but if they’re accompanied by their parents, they do not dare do it” (adult female, Lake Village E1).

In the absence of supervision or accountability, it seems relatively common for all age-groups to relive themselves outside. Focus group participants also identified the need for farmers to relieve themselves outside while working in their fields, where there are no sanitation facilities, as described by a 15-year-old male student from the youth group in Lake Village W:

The fields, the majority of the time, we defecate after we urinate because there… there is no toilet. (15-year-old male student, Lake Village W)

Despite the rules against open defecation, it appears not to be an uncommon occurrence in these villages, especially those along the lake. In all cases, the rationale for these rules had to do with keeping surface water clean for drinking or other uses. At no point did focus group participants mention that the breaking of these social rules contributed to the transmission of and risk for schistosomiasis.

### Finding 2: individual- and community-level prevention behavior.

In many villages, focus group participants across gender and age demographics described their efforts to limit their exposure to schistosomiasis and reduce their risk of infection by reducing water contact during midday heat. A middle-aged woman from the River Village typified this description in reporting:

When it gets hot, really hot, we do not enter [the water] because we say that it is at this time that bilharzia [schistosomiasis] enters the body. (54-year-old female gardener, River Village)

She noted that the norm in her village is to reduce water contact behavior during high-risk times of day to reduce the risk of infection. In another village on the lake, one woman discussed a similar pattern of behavior, where people went to the lake to bathe at the lower risk times of day:

Now at 1 pm, if you go to swim there, the parasite can get into your body… that’s why sometimes we have the habit of bathing in the morning… or in the evening, around Takussan [5pm prayer time] or later. (28-year-old female gardener, Lake Village E1)

These reports of avoiding water contact during times of high heat were articulated across focus group discussions in all villages and across all age and gender groups (Supplemental Table 2). One man in Lake Village E1 mentioned that many adults in his village have successfully stopped bathing in the lake altogether:

Bathing, not everyone does it [at the lake] because there are people who have not bathed at the lake [in] ten years. There are people who went ten years without going to the lake to bathe. (36-year-old male gardener, Lake Village E1)

In Lake Village W, focus group participants in both the men’s and women’s groups described a village-level effort to protect people from high-risk water contact. The chief of this village, who was one of the participants in the men’s group, spoke of a written policy that had been developed and agreed upon by a small group of village leaders to protect people from schistosomiasis. This policy outlined acceptable uses of the water access points. The village chief kept a handwritten record of this policy (Supplemental Figure 1). It outlines fines to be assessed to those violating acceptable use rules.

Focus group participants in this village also discussed imposing bathing schedules, or specific times of day that it was considered safer, relatively speaking, to bathe or perform other water contact activities. These schedules were shaped by the local perception that risk of infection was elevated around midday:

Now the hours during which we bathe, now we have chosen the schedules… because having the disease at these times is very easy... like noon, like noon, when it is hot. (47-year-old male gardener, Lake Village W)

An elderly participant in the women’s focus group in this village clarified that the policy was in place and enforced in the past but less so in the present. She said:

There was a system in place. It was better. The village chief was the guardian here… anyone who enters [the water], he tells you not to enter… there was a moment when the lake was so protected that bilharzia was [nearly] eradicated. (68-year-old female gardener, Lake Village W)

The same woman spoke at more length about the same program, identifying the importance of the chief’s leadership and influence:

We know that we were the first [village]… in the prevention against bilharzia… It [the village’s prevention effort] existed but it no longer exists. When we did it, it was deployed as an effort, the village chief who is here, if everyone was like him… the bilharzia would have moved away. (68-year-old female gardener, Lake Village W)

Whereas the chief was instrumental in leading and enforcing this village-level effort to eliminate schistosomiasis from the local water points, this woman’s words also highlight the challenge of engaging the entire community in the prevention efforts. In the absence of effective enforcement and widespread buy-in across the community, infected persons who persistently visit the local water points can single-handedly maintain the circulation of the parasite through its environmental stages. Whereas the chief’s leadership remains an important part of this village’s fight against schistosomiasis, this woman’s insight emphasizes the limits of a single person’s influence in behavior-based prevention of schistosomiasis.

The participants suggest that knowledge of the environmental risk of schistosomiasis has translated into preventive behavior in these villages, at both the individual and community levels. With good leadership, villages can develop and enforce rules that limit exposure to schistosomiasis. However, even in Lake Village W, where this was achieved, the program appears to have been short-lived without widespread community engagement and without strong perceived benefits or strong perceived risk of adverse impacts from infection.

### Finding 3: unavoidability of exposure.

Despite having translated high levels of knowledge into strategies for avoiding water contact, men, women, and children across all four villages cited the lack of alternatives as the reason they continue to complete the most common water contact activities in nearby surface water. The importance of subsistence agriculture to local livelihoods partially explains the persistence of water contact behavior despite widespread awareness of schistosomiasis. Most families in these four villages cultivate crops (Table 2), and those who spend time working canals and gathering water for their gardens are likely regularly exposed to schistosome parasites. Because of this, it is difficult for many farmers to eliminate contact with water, as stated by one farmer in Lake Village E1:

As long as you cultivate, you are in contact with water. (36-year-old male farmer, Lake Village E1)

A participant from the youth focus group in Lake Village E2 noted that farmers tend to bathe in the lake at the high-risk time of day after spending the morning in their fields:

There are people that report that it [schistosomiasis] comes out at certain times… that is to say, when it is hot. At this time, most field owners have finished their work and most of them go to the lake to bathe. (18-year-old male gardener, Lake Village E2)

Beyond agricultural fields, other occupational tasks, such as fishing, often require contact with water:

You know, we all go fishing. We are fishermen… we go to the lake… We are going fishing, but sometimes when you sit on the sides of your canoe in the lake and you have your feet in the water, bilharzia can enter your body. There is also a fish, which… when we fish it, we have to get out of the canoe to wade and walk. Here again, bilharzia can enter your body. (68-year-old male gardener, Lake Village E2)

Emergent vegetation like cattail reeds (e.g., *Typha* spp. and *Phragmites* spp.) are often harvested from the lake for use as material for roofs, fences, or floor mats. Harvesting this vegetation requires wading into the standing water in which it grows, according to one adult man from Lake Village E2:

So since then [dam construction], we live with schistosomiasis. So far, we cannot get rid of it, because we are working in it [the water]. We do not have the means to have hard [concrete] rooms or something that would protect us from the lake, or to install faucets. You see right now where we are [under a straw canopy at the chief’s house], the roof is made of grass that comes from the lake. And we have to be there for at least an hour. And anything can happen to you there. It’s this that has caused the disease. (an adult male, Lake Village E2)

This participant refers to the rudimentary materials available for housing construction in his village, citing that people cannot afford the concrete constructions commonly used in larger and less isolated communities. This circumstance increases the dependence of this community on the natural resources in their immediate surroundings, and, thus, their contact with water. Men from villages on both the lake and the river spoke of the difficulty of avoiding contact with water:

That water, we cannot touch it. We cannot abandon it. If we abandon it, we will all become unemployed. (53-year-old male gardener, River Village)

This man sums up the seemingly inescapable tension of carrying out a subsistence livelihood dependent on freshwater in a landscape with high risk of schistosomiasis transmission. He acknowledges that the water should be avoided to keep from becoming infected with schistosomiasis. If he could avoid it, he would, but that would require him to halt the activity that enables him to earn his livelihood and feed his family.

Although adults maintain some baseline exposure to surface water through their and occupational activities, it may not be so easy for women to reduce water contact behavior, even in the presence of safe alternative water sources. One man in Lake Village E1 suggested that it may be difficult to completely eliminate their exposure:

…but women, because they go there to do the laundry and other things, they have no choice. (43-year-old male farmer, Lake Village E1)

From this man’s perspective, even with widespread access to piped water sources in Lake Village E1 (Table 2), and knowledge of schistosomiasis risk in the lake, water contact activity at the lake likely persists for women. Similarly, exposure among children, the demographic group at highest risk of infection, was described as equally—if not more—inevitable. Although some children, especially girls, perform chores at the river, much of their exposure comes from playing in the water. For example, an elderly woman from Lake Village W described how much kids in her village play in the water:

Children only know swimming. You know, they are happy when they bathe. It’s a kind of a pool. They like that. In this moment, if you go to the lake, you find children in the process of bathing. It’s this that… gives us bilharzia. (68-year-old female gardener, Lake Village W)

Adult participants in focus group discussions often emphasized the importance of protecting children from schistosomiasis. Some mentioned that they forbid their children from going to the water points at hot times of day. They also said that many parents do not enforce rules about water contact with their children. Following this thread, the same woman from Lake Village W continued:

Nobody now controls their children. No one controls anything anymore. They [the children] are there [at the water access point]. Every child who pees, he pees blood. Because now we sit and let the children be. They will bathe. Nobody holds them back. If we had said, if they have given up bathing only, bilharzia would leave here. Because she [schistosomiasis] had left here. But she [schistosomiasis] came back. Because we let the children swim, swim, swim. (68-year-old female gardener, Lake Village W)

Even so, for those parents who did make rules regulating their children’s contact with water, adult focus group participants described the difficulty of enforcing rules. With these hurdles to reducing exposure among kids along with the health-livelihood trade-offs that many adults face, it is understandable that the local sentiment of schistosomiasis being an unavoidable artifact of the disturbed landscape has become pervasive. A 60-year-old female gardener from Lake Village W articulated this sense of inescapability:

It [water contact]’s not good for you. It’s not good. But we have no choice… Because we, we are near the lake. If you say that I will not do it [go to the lake] … it is certainly not possible. It cannot happen like this. Fortunately, we pray for ourselves and he [Allah] gives us medicine [praziquantel]… But we cannot protect ourselves… we are not easy to protect. (60-year-old female gardener, Lake Village W)

This woman expressed appreciation for the medicines that were occasionally administered to cure the disease, but did not speak of them as panacea as she was aware that reinfection was likely because of persistent contact with parasite-infested water. Because these villages are situated so close to the lake, avoiding water contact is not feasible. Villagers are resigned to chronic exposure: the disease persists in humans because the parasite and its intermediate snail hosts thrive in the environment on which villagers rely for daily needs. They lack alternatives despite lucid awareness of the risk.

## DISCUSSION

Local perspectives revealed during focus group discussions in these four villages highlight the importance of accounting for local context when designing schistosomiasis control and elimination programs in high-transmission settings. These findings demonstrate both barriers and opportunities to control. Importantly, we learned that people in this particular setting view schistosomiasis as an unavoidable fact of life, with few alternatives for avoiding occupational or domestic contact with water, despite their best efforts to minimize exposure. Yet, local recognition of epidemiologically relevant elements of the parasite life cycle have translated into behaviors meant to reduce risk, representing an opportunity for engaging local communities in schistosomiasis control efforts that complement MDA programs. In the following paragraphs, we discuss this tension between health and livelihoods, and what implications this and the opportunities for community engagement have for the design of schistosomiasis control and elimination programs in persistent hot spots.

### Tensions between health and livelihood.

The findings of this study highlight how local socio-environmental circumstances contribute to persistent transmission and high rates of infection even in the presence of MDA programs. Although focus group participants knew to avoid water contact to prevent exposure, high prevalence of infection and high dependence on water contact sites for daily living make clear that behavioral adaptations do not eliminate exposure outright. When forced to choose between economic well-being and avoiding the subtle morbidity of schistosomiasis, people inevitably choose the more immediate need for subsistence and risk the long-term health consequences of schistosome infection that accumulate slowly over time.

The economic needs that keep people in contact with water are often coupled with conditions of poverty—such as unsafe water supplies, inadequate sanitation, or poor housing conditions—that insufficiently isolate pathogens from their human hosts.^2^ Under these conditions, infected people easily perpetuate transmission by introducing a new generation of parasites into the environment through parasite-laden urine and feces, increasing infection risk for those who regularly use surface water. Without alternatives for isolating parasites from the environment, conditions of poverty increase parasite transmission and vice versa.

These feedbacks between disease and poverty may become self-reinforcing, such that those without the economic resources to treat or prevent illness will suffer higher burdens of disease and losses in productivity as a result, entering a vicious cycle known as a disease-driven poverty trap (DDPT). Disease-driven poverty traps are thought to be present in underdeveloped economies where 1) income is generated via resource-dependent livelihoods and people rely on their physical ability to exploit natural resources, and 2) the burden of infectious diseases in the population is high, such that, in causing disease, pathogens commandeer the human resources that would otherwise go toward income generation.^4^ Because health is considered a form of human capital, forming the basis of labor for generating income and alleviating poverty, reductions in cognitive development, and labor productivity resulting from infection can take a toll on the ability to develop human capital and earn a livelihood across the life course, potentially perpetuating poverty.^45^ Theoretical work on the subject suggests that infectious diseases may be an important barrier to economic development.^46,47^

Findings from this study use local perspectives to demonstrate the links between poverty and exposure to schistosomiasis, partially supporting the notion that schistosomiasis transmission in rural agricultural areas facilitates DDPT formation. Rural, subsistence livelihoods increase exposure to parasites embedded in the environment. Exposure becomes unavoidable because few alternatives are available for the daily tasks that put people in contact with water. So, as long as people undertake occupations that keep them in contact with water, they will remain infected.

Our qualitative data do not speak directly to the disease-to-poverty component of the feedback loop, but the literature suggests that this increased exposure will take a toll on cognitive development in children and labor productivity in adults and continue to hinder healthy development in a cycle of persistent poverty.^11,48^ The poverty trap may be interrupted by allowing people to pursue agriculture in an environment whose water sources are free of the parasite and its intermediate hosts. If the risk of acquiring infection from the environment is reduced, the unfortunate trade-off between health and livelihoods lessens, too. In addressing the environmental source of schistosomiasis infection, fewer human capital sacrifices—in the form of health, nutrition, educational attainment, and labor productivity—may facilitate economic development unhindered by this NTD.

### Opportunities for community engagement.

In our study, the frequent mention of increased risk of schistosomiasis infection at midday reflects published knowledge of eco-epidemiological dynamics of schistosomiasis transmission to humans from snail intermediate hosts. Snails are known to shed parasites in daily cycles, which peak around noon for human schistosomes.^49,50^ Although focus group participants did not articulate knowledge of the snail-borne nature of schistosomiasis transmission, they did recognize the diurnal patterns of risk and reported risk-mitigating behavior. An intervention based on a similar scheme of risk mitigation was conducted among irrigation workers in central Sudan, demonstrating significant reductions in reinfection rates when workers limited their water contact to the early morning hours.^51^ The local knowledge and risk perception revealed through this study could be translated into community-driven interventions that complement existing MDA programs.

In contrast to the top-down MDA campaigns that are typical in current schistosomiasis control programs, our findings suggest that local communities are important and underused resources in the fight against schistosomiasis. Engaging local populations and empowering action to reduce the burden of schistosomiasis could play an important role in reducing rates of reinfection following MDA campaigns in persistent hot spots. To date, few schistosomiasis control studies focus on the role of the community or use existing local knowledge. The few that have, however, used community input in diverse ways. Codesigned sanitation facilities and laundry platforms were used to reduce contamination behavior and exposure, respectively.^52^ A community-identified need for an environmental approach to snail control involved a local irrigation council in the removal of vegetation from canals to reduce snail habitat.^53^ Another study engaged religious teachers in behavior change interventions.^54^ Our findings further reinforce the importance of integrating community participation into the design and implementation of prevention strategies. Such community-informed interventions have the potential to improve long-term and sustained implementation of schistosomiasis control and reduce transmission between MDA campaigns in locally appropriate and acceptable ways.

### Implications for disease control.

In this section, we discuss the implications of our findings for schistosomiasis control considering the recent history of schistosomiasis control campaigns prioritizing MDA. In persistent hot spots and settings where resource-dependent livelihoods make it impractical to eliminate water contact, MDA campaigns are unlikely to achieve the prevalence and intensity targets outlined by the World Health Organization.^18,19,55–57^ Supplementing MDA campaigns with environmental strategies such as snail control and WASH provision that reduce risk of (re-)infection has the potential to have a high impact in these settings. Strategies that decrease environmental risk of infection will improve the outcomes of MDA campaigns: reducing existing infections, preventing new ones, and increasing the likelihood of local elimination.^58^

Although piped water provides an important alternative to the water access points where people acquire schistosomiasis, it is not likely to eliminate exposure outright. This is apparent in two of our study villages (River and Lake E1), where piped water access is nearly 100% but where schistosome infection prevalence is not substantially lower than the two villages with much lower access to piped water (Lake W and Lake E2; Table 2). Even if many HHs in a village have access to a safe source of water, it is still relatively common to visit the river or lake for chores, such as doing laundry (Table 2). A similar persistence in water contact in the presence of piped water sources has been reported in other settings.^58^

In our study setting, there were several explanations for persistent water contact despite piped water access. Some adult women focus group participants told us about the social value of going to the river or lake to do laundry. Others declined to pay the modest fee for using a piped water source when water was freely available from the lake or river. This was especially true for larger tasks such as washing livestock, for which it was impractical to use piped water. In addition, piped water sources in some villages were only available for a couple of hours per day, such that people formed long lines to retrieve water in the brief windows when it was available. In these ways, surface water can be viewed as more affordable and convenient, even if it is hazardous.

These local nuances emphasize the importance of understanding local context when designing disease control and elimination programs. The failure of environmental interventions in public health may reflect poor understanding of how people relate to their environment, including how and why behaviors related to exposure and disease are constrained by circumstances.^59^ In understanding relevant circumstances and constraints in context, policies can be designed to appeal to the common sense of the intended beneficiaries rather than the enactors of a policy.^60^ Such efforts are more likely to mobilize and empower the local action and buy-in required to gain and sustain control of schistosomiasis in persistent hot spots.^60^

With a wide range of strategies available to control and eliminate schistosomiasis, local circumstances should inform which strategies are used and in what combination. It is clear that MDA has been effective in many settings, resulting in meaningful reductions in prevalence and intensity of infection across target populations.^61^ In the settings where schistosomiasis remains a problem despite well-implemented MDA programs, it is critical to supplement MDA with other strategies to achieve disease control and, eventually, elimination targets. Which interventions complement MDA—whether chemical^17,33,62^ or biological^31,32,37^ snail control, provision of water and sanitation infrastructure,^34–36^ or behavior change interventions^51,54,63^—should be informed by the social and environmental factors at play in a given setting in close consultation with communities themselves. Prioritizing community engagement in integrated strategies will improve the likelihood that they are adopted and sustained over time.

### Limitations.

It is important to note that the findings of this study are based on a small number of participants in a small number of villages using a nonrandom recruitment process and self-reported data. Thus, we cannot consider our findings generalizable to the regional population.

In addition, our conclusions have necessarily been drawn from self-reported perceptions and behaviors, which may be subject to bias. Because focus group participants were aware of the research team’s purpose of studying schistosomiasis in the village, it is possible that responses given during focus group discussions were subject to social desirability bias. The disperse nature of water contact behavior, often covering multiple water access points and agricultural fields, made it difficult to verify reported behaviors with direct observations. The age range of participants differed across groups and villages, which may have introduced some selection bias. Notably, participant age did not exceed 41 years in women’s groups in Lake Villages E1 or E2. Similarly, elderly participants were missing from the men’s groups in the River and Lake E1 villages, with a maximum age of 53 years. Without elder representation in these groups and villages, it is possible that we did not capture memories of past interventions similar to those identified in Lake Village W particularly in Lake Village E1, where the maximum age across all focus group discussions was 49 years.

It is also important to note that because of the significant disease burden, the lower basin of the Senegal River has received a great deal of research attention since the late 1980s. The knowledge expressed in the focus groups may be the result of repeated interactions with researchers and public health officials addressing the now endemic situation. These findings might not reflect general knowledge in villages that have never been part of scientific projects or the National Schistosomiasis Control Program. Although our findings suggest that local people have been educated by their experience living in this setting, it is important to acknowledge that part of that experience has been interactions with authorities who have shared knowledge with local people. This does not diminish the existence of the local knowledge, but rather suggests that this influence becomes part of the local knowledge. If anything, the high levels of local knowledge we report here illustrates the value of awareness and educational campaigns.

Finally, it is possible that key informants’ knowledge of our intentions to study schistosomiasis in their communities may have biased them to recruit volunteers who were more knowledgeable than the average person. Although not every individual in every focus group was equally knowledgeable about schistosomiasis, the local contacts’ impressions of the research team and its objectives may have resulted in us concluding that villagers are more knowledgeable on average than they are. However, even if our participants represent a more knowledgeable subset of the village population, their knowledge of schistosomiasis risk and their capacity to assume leadership in their communities are noteworthy starting points for community engagement.

## CONCLUSION

This study reveals that rural residents of northern Senegal, a region hyperendemic for schistosomiasis, have a keen awareness of schistosomiasis risk in their surroundings. They reported acting on their knowledge to prevent infection, even demonstrating capacity to organize at the community level. These findings point to the importance of engaging communities in schistosomiasis control programs, in addition to relying on top-down treatment campaigns. The perceived unavoidability of schistosomiasis exposure—and all its insidious consequences on health and human capital—despite prevention efforts at the individual and community levels suggests the presence of a vicious cycle of poverty and disease. With such ostensibly intransigent transmission in this setting, environmental interventions that reduce the risk of reinfection, given the local dependence on surface water, become a necessary complement to MDA.

By gathering the perspectives of local people, this study has identified inherent limitations of control programs that rely predominately on MDA in hyperendemic regions, as they reduce morbidity but do not prevent reinfection. Rural livelihoods facilitate exposure to schistosome parasites in a disturbed landscape, such that infection has become an unavoidable fact of life. Because chronic infection caused by continued exposure to contaminated water inevitably reinforces the conditions leading to persistent poverty, integrated schistosomiasis control strategies are needed to break the cycle of disease and poverty. Whereas the World Health Organization already recommends the integration of MDA, snail control, and WASH interventions for schistosomiasis elimination, this approach becomes doubly important for high-transmission settings that also experience poverty trap conditions. Through community engagement, the implementation of these integrated strategies is more likely to be adopted and sustained over time.

## Supplemental files

Supplemental materials
