# Supplementary material for: Unavoidable Risks: Local Perspectives on Water Contact Behavior and Implications for Schistosomiasis Control in an Agricultural Region of Northern Senegal
Source: Am J Trop Med Hyg. 2019 Aug 26;101(4):837–47. doi: 10.4269/ajtmh.19-0099 (PMC6779182; doi:10.4269/ajtmh.19-0099)
Supplement: Supplementary file 1 [file tpmd190099.SD1.docx]

**Supplemental Information.**

**Supplemental Appendix S1. Quotes supporting each finding, stratified by geographic, age and gender groups**

**Table S1**. Representation of quotes across geographic, age and gender groups for Finding 1. High local knowledge of schistosomiasis risk

|  | **Men** | **Women** | **Youth** |
| --- | --- | --- | --- |
| **River** | “It is there [at the river] that bilharzia enters your body. If you are told something else, know that you are cheated. It is there that you feel bilharzia enter squarely into your body.” – gardener, 53  “Participant 1: It is during the periods of high heat, from 1pm to 2pm everyone comes out here to bathe. Participant 2: It’s the same as what he has told you. It is when it is hot that the schistosomiasis spreads more easily… Yes that’s the way it comes out and gets left in the water. Now if you are used to bathing in the late afternoon, you have itches after getting out of the water. And when the skin itches like that it means that you are being infected with bilharzia.” –gardener, 21 and gardener, 53 | “Because during the rainy season it is hot. And it is said that during the rainy season, when the sun gets hot, that schistosomiasis comes out... And we remain soaked in the water while bathing. You can see a woman there in the morning until 2pm doing laundry. She stays at the river getting in and coming out again.” –trader, 23 |  |
| **Lake-W** | “When it is hot, it is very easy to catch schistosomiasis.” –gardener, 47  “You see someone that comes here [to the village] to pass a month of holiday here, and if he returns, he has bilharzia… I have a nephew who a few days ago was asking for drugs for bilharzia because he was peeing blood. And yet, he had just come here to visit for Gamou [a holiday]. Just Gamou. Two days, three… where did he get bilharzia?... He, he entered the lake. He took a few steps in the lake, but he did not spend the day here. Now… he is the one who says he has bilharzia.” –gardener, 48  “The water with which you irrigate, you draw it from the canal which comes from the lake. That’s how you spend the day even if its very hot… and about bilharzia, she walks during the day.” -gardener, 48 | “Bilharzia, it is everywhere in the lake where we bathe.” – gardener, 68  “And when it’s hot, you see that the bilharzia comes out. Now if you walk in the water, it enters your body… If you swim in it too, it catches you.” – unidentified participant  “Most of the time, the kids, they enter the lake to bathe. This bountou [water access point], can you see them? They are in the water in the process of bathing… To bathe there like that is what gives them the most bilharzia.” – gardener, 68  “It is noted that bilharzia, the fact that children bathe in the lake, they are the most affected… Most of the time, the kids, they go to the lake to bathe. This bountou [water access point], they are in the water in the process of bathing. And this hour on, nothing is good. To bathe there like that is what gives them the most schistosomiasis.” – gardener, 68  “The fact that we walk in the water, according to those who pay attention, it is here that it is easier to catch it. And there is a place [where]… everything you touch is water, you more easily have bilharzia.” –gardener, 68  “It [going to the bathroom there] is not non-existent, but it is forbidden” – gardener, 68 | “To have it [schistosomiasis] in the lake is easier… Because we bathe most often in the lake… We go there most of the time.” - male student, 15  “The lake is close to the village. At any moment, the children go back and forth [to the lake] even if the sun is hot, hot.” – unidentified participant  “The fields, the majority of the time, we defecate after we urinate because there… there is no toilet” – male student, 15 |
| **Lake-E1** | “Places that are the shelter for bilharzia are places that we are accustomed to frequenting. These places are the bounts [water access points], [the] lake where we bathe frequently.” – unidentified participant  “I think it’s at the level of the bounts [water access points that schistosomiasis risk is highest] ... because what I saw as a case of schistosomiasis in children... because they go frequently to the bounts... if you tested them, I think you would have 100%.... because all the time they are at the lake.” -farmer, 49  “As long as you swim, where you enter the canals to draw water from it with cans, it is at this moment when you are the most exposed.” – unidentified participant | “You know the sun, the hotter it is, the more powerful the parasite is... it comes out more... because around noon, 1pm, that's when the parasite is more open... Now at 1pm, if you go to swim there, the parasite can get into your body.” - trader, 28  “Sometimes the kids, they go there. Sometimes we see them there peeing or pooping… but each time we see them, we forbid them from doing it… because we drink that water and we bathe with it… Again, we want to preserve our bodies… that’s why each time we see them, we forbid them.” – trader, 28  “At all the [water access sites], you know sometimes the children, they go there [to go to the bathroom] … The kids, when they are alone, they do what they want… but if they’re accompanied by their parents, they do not dare do it” – unidentified participant | “Because when you bathe, bilharzia enters your body.” –female student, 12  “Schistosomiasis occurs when the sun is warm.” – male student, 12 |
| **Lake-E2** | “With schistosomiasis, as long as you put your feet in the water, all [water contact] places are the same… As long as you put your feet in the water, you cannot escape.” – gardener, 38  “The risk of getting infected with schistosomiasis is more common there where you put your feet. The more you frequent the water, the more the risks are.” – unidentified participant  “When you come in [the lake], you risk being infected by the parasite that can live in you.” – unidentified participant  “You stay in the water and the water comes from the lake. This water can stay there for a long time. So when you come in, you risk being infected by the parasite that can live in you.” – unidentified participant  “If it’s the water access point that has trees next to the water, sometimes, there are careless adults… who find shelter to do it [go to the bathroom] there” – gardener, 38  “Sometimes in your field, you may want to urinate or defecate. There, you go somewhere in a small corner… We cannot do anything… because there is no toilet” – gardener, 57  “If you turn around and you don’t see anyone, you pee, you poop” – gardener, 68 | “People know that the disease comes from the lake.” – trader, 32  “It’s between 11am and noon that it is most susceptible to be infected with bilharzia, washing yourself, it can penetrate you without you feeling it.” -homemaker, 28  “If you want to go to the bathroom, we take the water from the canal and we hide behind to do what we need… If someone sees you doing your thing [going to the bathroom] in the lake, he tells you not to do it because you drink the water” -homemaker, 28 | “During the heat, we can become infected with bilharzia if we go to these three water points around midday and 1pm… during the heat… when the sun is at its zenith.” – gardener, 15  “There are times when there is less risk. Right now, for example, the water is not hot. The microbe is not circulating. But around 2pm, 1pm to 3pm, the risks of the disease are there. We know that people go there at these times.” – 16-year old male student  “Sometimes when you go to the bank [of the lake] during the heat, you are infected with schistosomiasis.” –male gardener, 15 |

**Supplemental Table S2**. Representation of quotes across geographic, age and gender groups for Finding 2. Preventive behaviors.

|  | **Men** | **Women** | **Youth** |
| --- | --- | --- | --- |
| **River** | “There are parents who make arrangements and ask their children to not go to the river. Some parents take care of that, but others do not.” – gardener, 53 | “When it gets hot, really hot, we do not enter because we say that it is at this time that bilharzia can enter the body… It is forbidden for anyone to enter.” –gardener, 54 | “When it is hot, you don’t bathe. When it is hot, you do not swim, don’t you?” – unidentified participant |
| **Lake-W** | “Now the time during which we bathe, now we have chosen the schedules... because having the disease at these times is very easy… like noon, like noon, when it is hot.” – gardener, 47  “Now the one whose child goes to the lake, you ask him to get out of the lake” –gardener, 47 | “There was a system in place. It was better. The village chief was the guardian here… anyone who enters [the water], he tells you not to enter… there was a moment when the lake was so protected that bilharzia was [nearly] eradicated.” –gardener, 68  “We know that we were the first [village]… in the prevention against bilharzia… It [the village’s prevention effort] existed but it no longer exists. When we did it, it was deployed as an effort, the village chief who is here, if everyone was like him… the bilharzia would have moved away.” –gardener, 68  “At this time, it [water contact] must be stopped... It is necessary to stop, in any case to avoid the water... until it becomes cool.” -unidentified participant  “The adults, none of them bathe in the lake. You go back to your room, to your house, it’s over the toilet that you take a bath… you go in your shower to take a bath.” –gardener, 68 |  |
| **Lake-E1** | “Bathing, not everyone does it [at the lake] because there are people who have not bathed at the lake [in] ten years. There are people who went ten years without going to the lake to bathe.” –farmer, 36  “Many do not go to the lake anymore because of that [to avoid the disease] … Yes, it [attendance at the lake] has dropped for adults.” –farmer, 43  “At other water points [the fields and the gardens], we wear shoes, boots and sometimes we are not very wet... when you have shoes, you can be there from morning to night without getting wet... If you only stay in the field, it will not be easy for you to be infected with schistosomiasis.” – unidentified participant  “But all the adults who have the choice, like us… who can stay away [from the lake] for six months without going to the lake, do not go there.” – farmer, 36 | “The children, the freedom they had, the total freedom that they enjoyed… now it has diminished… Now certain free times… in this moment, there are some, when they don’t go to school, we give them cattle to graze… the freedom he had to go to the river… now he does not have it anymore … All that, it is to occupy the children… so they do not have too much freedom.” –trader, 28  “Now at 1pm, if you go to swim there, the parasite can get into your body… that’s why sometimes we have the habit of bathing in the morning… or in the evening, around Takussan [5pm prayer time] or later.” –gardener, 28  “Our children… towards Takussan [5pm prayer] … that’s when they go to the lake, but those in the middle, they are forbidden. They do not go… it’s just at home that they take a bath.” –hairdresser, 27 |  |
| **Lake-E2** |  | “My children and I have seen this [visits to the lake] decrease… because I forbid it for those who want to go there... at certain times.” –trader, 32 | “There are times when there is less risk. Currently, for example, the water is not hot. The microbe does not circulate.” –male student, 16 |

**Supplemental Table S3** Representation of quotes across geographic, age and gender groups for Finding 3. Unavoidability of exposure.

|  | **Men** | **Women** | **Youth** |
| --- | --- | --- | --- |
| **River** | “That water, we cannot touch it. We cannot abandon it. If we abandon it, we will all become unemployed. Even if we would die, we would stay in contact with it.” –gardener, 53  “You know kids, you cannot control them all the time.” –gardener, 53  “There are even parents who allow their children to go to the river but not during periods of high heat.” –farmer, 48 |  |  |
| **Lake-W** |  | “Children only know swimming. You know, they are happy when they bathe. It’s a kind of a swimming pool. They like that. Right now, if you go to the lake, you find children in the process of bathing. It’s this that… that gives us bilharzia.” –gardener, 68  “Nobody now controls their children. No one controls anything anymore. They are there. Every child who pees, he pees blood. Because now we sit and let the children be. They will bathe. Nobody holds them back. If we had said, if they have given up bathing only, bilharzia would have left, because it had left here. But it has come back. Because we let the children bathe, bathe, bathe. The time they should bathe, because bathing, if you begin at 5pm or 6pm, if you bathe [then], there is not a problem… if you start at 8am or 9am, there is not a problem. But at this time [midday], if you bathe, bilharzia can catch you.” – gardener, 68  “It’s not good for you. It’s not good. But we have no choice… May God preserve us… Because we are near the lake. If you say ‘I will not do it’… it is certainly not possible. It cannot happen like this. Fortunately, we pray for ourselves and we are given medicines… But we cannot protect ourselves… we are not easy to protect.” – 60-year old gardener | “The lake is close to the village. At any moment, the children go back and forth [to the lake] even if the sun is hot, hot.” – unidentified participant |
| **Lake-E1** | “As long as you cultivate, you are in contact with water.” –farmer, 36  “…but women, because they go there to do the laundry and other things, they have no choice.” –farmer, 43 | “…but the children, you know they’re stubborn… sometimes they wait until noon to go swimming.” –gardener, 28  “The lake, it is more frequent [to catch schistosomiasis] … the hours in which we go there… you can go there in the morning or afternoon, when the sun goes down. But we, we don’t do that. We wait until noon, 1pm to go to the river. And its at this hour that schistosomiasis rises.” – gardener, 28 |  |
| **Lake-E2** | “You know, we all go fishing. We are fishermen… we go to the lake… We are going fishing, but sometimes when you sit on the sides of your canoe in the lake and you have your feet in the water, bilharzia can enter your body… There is also a fish, which… when we fish it, we have to get out of the canoe to wade and walk. Here again, bilharzia can enter your body.” – gardener, 68  “So since then [dam construction], we live with schistosomiasis. So far, we cannot get rid of it, because we are working in it [the water]. We do not have the means to have hard rooms or something that would protect us from the lake, or to install faucets. You see right now where we are [under a straw canopy at the chief’s house], the roof is made of grass that comes from the lake. And we have to be there for at least an hour. And anything can happen to you there. It’s this that has caused the disease.” – unidentified participant  “I know that here, whether you are a farmer or a fisherman or whatever your job, you cannot escape bilharzia… As long as you enter the lake, you cannot escape bilharzia.” –gardener, 38  “And whatever your activity, as long as you’re in contact with the water, you cannot escape bilharzia.” – 68-year old gardener  “With schistosomiasis, as long as you put your feet in the water, all places are the same. All places are the same. As long as you put your feet in the water, you can not escape.” – gardener, 57 |  | “There are people that report that it [schistosomiasis] comes out at certain times… that is to say, when it is hot. At this time, most field owners have finished their work and most of them go to the lake to bathe.” – male gardener, 18  “There are people working there [at the lake]. They are obliged to go at these [high-risk] hours, that is to say, around 3pm.” – male student, 16 |

**Supplemental Appendix S2. Focus group discussion guide**

**Introduction (10 minutes)**

Good morning/day/evening. We are happy that you are able to share some time to discuss with us. Let’s begin by introducing ourselves.

[Moderator introduces himself, followed by each member of the research team and then the discussion participants]

All the topics we will be discussing today are important for understanding how schistosomiasis is transmitted in this village and others along the Senegal River and Lac de Guiers. This research is mainly to understand where and how people come into contact with water and become exposed to schistosomiasis. With this information, we hope to know where to target the fight against the disease. Your suggestions, ideas and comments about water contact in and around the village are welcome.

We would like that you talk about the different places where people come into contact with water and what they do there.

We would like to clarify that there are no bad answers in this discussion, only different points of view. Everyone should feel free to express your thoughts and opinions. Anyone can speak at any time, but we would like that only one person speaks at a time. There should be no side discussion. You are free to accept or not accept what is said. This work requires concentration, so please kindly put your phones on silent mode during the discussion. In the case that you have to take a call, please do so as quietly as possible and rejoin the group as quickly as you can. My role as moderator will be to lead the discussion, while giving you the opportunity to discuss amongst yourselves.

We are asking for your permission to record the discussion because all your responses are useful and we can’t write fast enough to be able to take note of everything. The recording will enable us to have the details you shared later. In place of your names, we are going to use numbers to ensure total confidentiality of the discussion. We will spend one hour in total. Before we begin the discussion, do you have any questions for us?

**Water Contact Places (25 minutes)**

We would like you to give all the places where people here come into contact with water. This could be a water point, an irrigation canal, a field or another place. At the end of the discussion, we would like that a volunteer to show us these places.

[The moderator explains what he means by water contact]

[For each identified place to which the notetaker has assigned a number, the moderator poses the following questions]

1. Can you tell us more about these places? [The moderator allows for open discussion and if necessary, poses the questions below for more detail]:
   1. How would you describe the environment at this place?
   2. Who goes to this place?
   3. How often do they go?
   4. What do they do there when they go?
   5. Can we go there to swim or go to the bathroom?
2. How do these places change in different seasons?
   1. Do these changes affect the different activities that you do?
   2. If yes, how?
   3. Do you think that people are more exposed to schistosomiasis by going to one water contact place over another?
   4. If yes, how do they know? Does this change their behavior so that they reduce their risk of infection?
   5. If yes, how?

**Final Remarks (5 minutes)**

We have held you for a long time to ask you questions. We would like to thank you for having shared with us your points of view about the ways of water contact. Before we finish, we would like to know if you would like to ask questions of us. [The moderator allows for open discussion].

Are there points that we have not raised that you consider important? [The moderator allows for open discussion]

We sincerely thank you for having taken the time to respond to our questions. Your responses will help us to better understand how human interaction with the environment influences the transmission of schistosomiasis in the village. With this information, we hope to be able to more effectively control the disease of schistosomiasis.

[The moderator reminds the group of the activity to follow and asks for a volunteer to accompany the team to visit water contact sites]

*
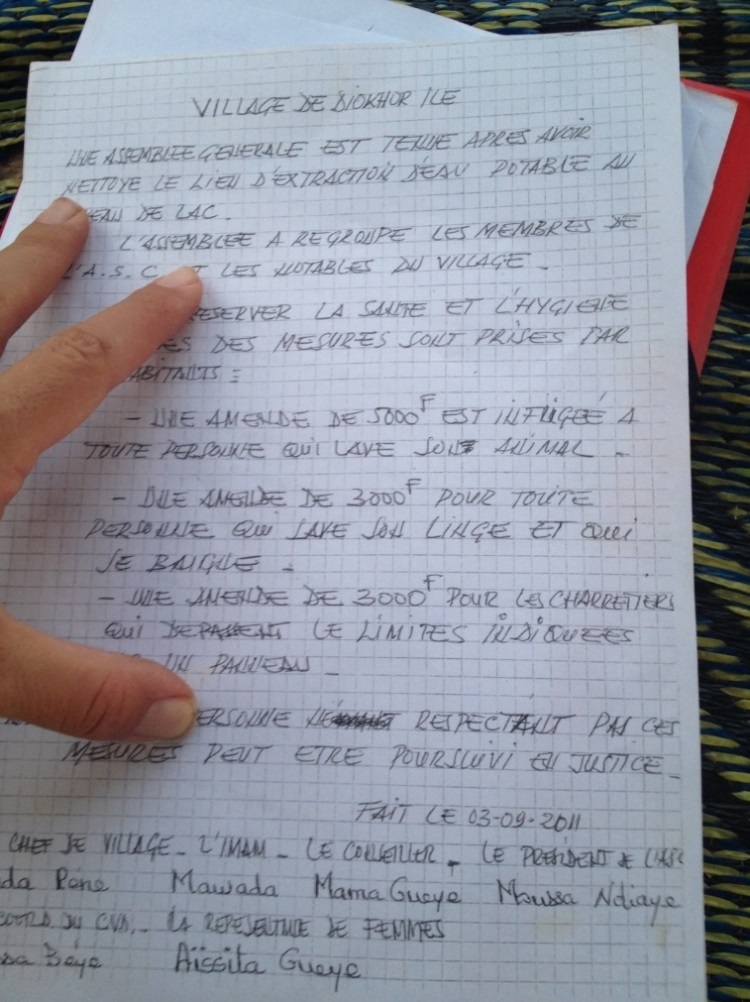
*

**Supplemental Figure S1.** Rules for acceptable use of water access points in lake village-W.
